# Short-Wavelength Sensitive Cone (S-cone) Testing as an Outcome Measure for *NR2E3* Clinical Treatment Trials

**DOI:** 10.3390/ijms20102497

**Published:** 2019-05-21

**Authors:** Alejandro J. Roman, Christian A. Powers, Evelyn P. Semenov, Rebecca Sheplock, Valeryia Aksianiuk, Robert C. Russell, Alexander Sumaroka, Alexandra V. Garafalo, Artur V. Cideciyan, Samuel G. Jacobson

**Affiliations:** Department of Ophthalmology, Scheie Eye Institute, Perelman School of Medicine, University of Pennsylvania, Philadelphia PA 19104, USA; aroman@pennmedicine.upenn.edu (A.J.R.); chpowers@sas.upenn.edu (C.A.P.); esemenov733@gmail.com (E.P.S.); rebecca.sheplock@uphs.upenn.edu (R.S.); vaks@sas.upenn.edu (V.A.); robert.russell2@uphs.upenn.edu (R.C.R.); asumarok@pennmedicine.upenn.edu (A.S.); garafalo@pennmedicine.upenn.edu (A.V.G.); cideciya@mail.med.upenn.edu (A.V.C.)

**Keywords:** cone, outcomes for therapeutic clinical trial, photoreceptor, retinal degeneration, visual thresholds

## Abstract

Recessively-inherited *NR2E3* gene mutations cause an unusual retinopathy with abnormally-increased short-wavelength sensitive cone (S-cone) function, in addition to reduced rod and long/middle-wavelength sensitive cone (L/M-cone) function. Progress toward clinical trials to treat patients with this otherwise incurable retinal degeneration prompted the need to determine efficacy outcome measures. Comparisons were made between three computerized perimeters available in the clinic. These perimeters could deliver short-wavelength stimuli on longer-wavelength adapting backgrounds to measure whether S-cone vision can be quantified. Results from a cohort of normal subjects were compared across the three perimeters to determine S-cone isolation and test-retest variability. S-cone perimetry data from *NR2E3*-ESCS (enhanced S-cone syndrome) patients were examined and determined to have five stages of disease severity. Using these stages, strategies were proposed for monitoring efficacy of either a focal or retina-wide intervention. This work sets the stage for clinical trials.

## 1. Introduction

Rare and previously incurable inherited retinal degenerations (IRDs) have recently been reconsidered as potentially treatable. With these advances in therapeutic possibilities has come the need to design clinical trials [1,2,3]. Monitoring safety depends on the expertise of ophthalmic and other medical clinicians using mainly standard examinations. Monitoring efficacy can default to time-honored clinical measures of vision such as visual acuity [4]. Such measures may be suitable outcomes for some IRDs but other disorders have retinal and visual mechanisms that are not routinely quantified.

**Figure 1:**
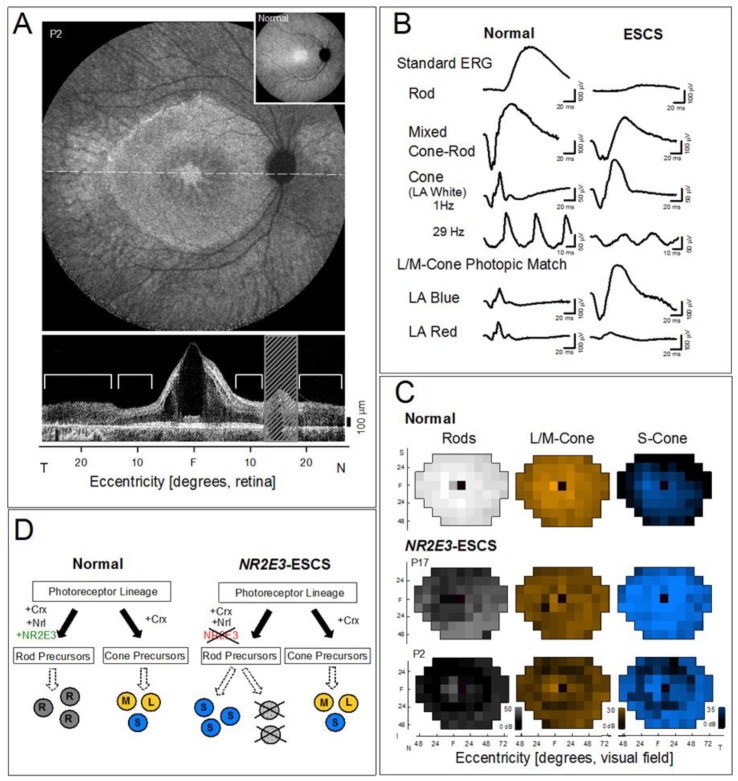
*NR2E3*-ESCS disease overview. (**A**) Fundus image and optical coherence tomography (OCT) in an *NR2E3*-ESCS patient. Upper: Infrared autofluorescence 55° wide fundus image shows spoke-like central changes indicating cystic abnormalities, adjacent annular hypo- and hyper-fluorescent changes within the vascular arcades, and surrounding retinal regions suggesting reduced retinal pigment epithelium (RPE) melanization and resulting choroidal visibility. Lower: OCT across the horizontal meridian showing cystic-schisis central changes and adjacent photoreceptor layer and RPE integrity but surrounded by thickened disorganized retina and a diminished RPE layer. Brackets above the described areas adjacent to the schisis. Hatched bar, optic nerve location. Inset, normal fundus image. (**B**) Electroretinograms (ERGs) in a representative normal subject (age 17) and an ESCS patient (age 13) under dark- and light-adapted adaptation conditions and using spectral stimuli. The standard ERG in the normal subject (upper left) illustrates a rod-mediated b-wave to a blue flash in the dark-adapted state; a mixed cone–rod maximal ERG to a white light flash, in the dark-adapted state; and cone ERGs in response to light-adapted 1 Hz flashes and 29 Hz flicker. The ESCS patient has a reduced response to blue flashes, dark-adapted; a large ERG signal to the white light, dark-adapted; and a typical difference in amplitude between the two light-adapted ERGs with flicker being far smaller in amplitude than the 1 Hz flashes of white light, which has a waveform reminiscent of the same stimulus in the dark. Light-adapted ERGs matched for L/M-cones in the normal subject (left) are mismatched in the patient (right) with a far larger ERG to blue than red flashes. Calibration bars (vertical, microvolts, µV; horizontal, milliseconds, ms) are at right and below the waveforms. (**C**) Color-scale maps of retinal sensitivity comparing normal subjects and patients. Left: Rod sensitivity (dark adapted, 500 nm); center: L/M-cone sensitivity (light adapted, 600 nm); right: S-cone (short wavelength) sensitivity (440 nm on bright yellow background), for a normal subject, and two *NR2E3*-ESCS patients (P17, P2). The scales for the results all have 31 intensity levels (key at lower right), with brightest corresponding to 50, 30, and 35 dB for 500, 600, and 440 nm respectively. Black indicates no detection of the stimulus. (**D**) Schematic of photoreceptor development and how lack of NR2E3 leads to excess S-cones. R: rod, L, M, S: long, middle and short wavelength sensitive cones. N: nasal, T: temporal, I: inferior, S: superior, F: fovea.

One of the IRDs being considered for therapeutic intervention is an autosomal recessive disease caused by mutations in the *NR2E3* gene; this condition was first identified three decades ago using visual function testing. After a history of clinical misdiagnosis as a form of retinitis pigmentosa (RP), X-linked retinoschisis, Stargardt disease, or stationary night blindness among other disorders, it was recognized as a specific entity. Diagnostic confusion was due to the constellation of features that could include nightblindness, visual acuity loss with cystic maculopathy or schisis (Figure 1A), pigmentary retinopathy and/or fleck-like lesions, peripheral retinoschisis, progressive visual field losses, and abnormal electroretinograms (ERGs). There was rod photoreceptor dysfunction, reduced L/M-(long/middle-wavelength sensitive) cone function but the unique feature of greater-than-normal (enhanced) vision of the least populous photoreceptor subtype, the S-(short-wavelength sensitive) cones [5,6,7]. Spectral ERG stimuli were used to dissect the mechanism of the abnormal patient waveforms and the conclusion was that the signals were from S-cones [6,8,9] (Figure 1B). Chromatic perimetry in the dark- and light-adapted states revealed little or no rod-mediated vision, reduced L/M-cone function and increased S-cone function (Figure 1C). We named this condition the enhanced S-cone syndrome (ESCS) [6,7,8,9]. The basis of the hyperfunctioning S-cones was postulated to be a developmental defect in cone cell differentiation causing greater numbers of these photoreceptors (Figure 1D), and subsequently leading to retinal degeneration and blindness [9,10]. The molecular cause was later identified as mutations in the orphan nuclear receptor transcription factor gene *NR2E3* [10,11,12,13]. The pathways that involve NR2E3 and determine photoreceptor cell fate continue to be investigated to the present day (for example, [14,15,16]).

The disease expression of ESCS patients with *NR2E3* gene mutations was recently reassessed, considering promising proof-of-concept murine research that could lead to clinical trials of treatment [13,14,17]. The conclusion was that the cellular target for human therapy would have to be the S-cone and a method to monitor S-cone function in the clinic was needed [13]. There is a long history of isolating S-cones, whether by electrophysiological or psychophysical methods, and these have been used to study individual color vision mechanisms in human subjects. Many of the methods tend to be more suited for a research environment than for a clinical trial population [18,19].

Chromatic adaptation, however, permits S-cones to be isolated not only in the visual science laboratory [20] but also in the eye clinic using computerized static perimetry. The method, color perimetry, has become a means for early detection of glaucoma, a common eye problem causing elevated eye pressure and optic nerve degeneration [21,22]. A blue stimulus projected onto a bright yellow background in various locations of the visual field allows sampling a wide or a localized area of vision in a relatively short time. In the present work, we take up the challenge to develop S-cone perimetry as an outcome measure for future clinical trials of treatment of patients with *NR2E3*-ESCS.

## 2. Results and Discussion

### 2.1. Comparison of Available Computerized Perimeters for S-cone Isolation

**Figure 2:**
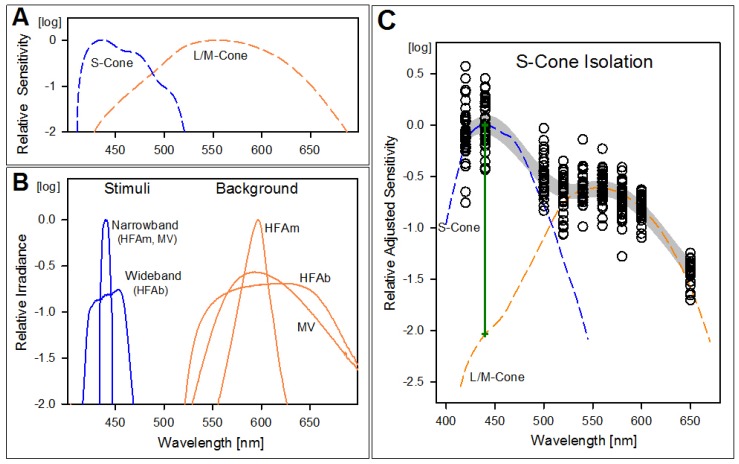
Isolation of S-cone responses. (**A**) Sensitivity of S- and L/M-cones mechanisms as a function of stimulus spectral content. They peaked at 440 and 555 nm, respectively. (**B**) Relative spectral content of stimuli and background lights. The stimulus beam filter used for machines HFAm and MV provide a narrower spectrum than the one in HFAb, and both are centered at 440 nm to maximize effectiveness for S-cones and minimize perception by L/M-cones (blue lines). The yellow background spectra for each machine are shown with orange lines: all content remains above 530 nm maximizing effectiveness to adapt L/M- cones and rods, and minimizing the adaptation of S-cones. (**C**) Sensitivity of normal subjects for stimuli of 9 different wavelengths when tested on the 100 cd.m^−2^ yellow background. Lower sensitivities on the longer wavelength range indicate a desensitized L/M-cone system as per effect of the bright yellow background. The S- and L/M-cone mechanisms (dashed lines) have been individually shifted vertically to match the subjects’ data on each range. The green line (2 l.u. high) indicates the amount of isolation (difference in S- and L/M-cone sensitivities at target center-wavelength) achieved with the test setup.

Three computerized static perimeters were studied in a cohort of normal subjects using short-wavelength stimuli and longer-wavelength adapting backgrounds to determine S-cone vision across the visual field using static perimetry [6,13,23]. This isolation method takes advantage of the difference in spectral sensitivities of the S- and L/M-cone systems, which peak at different wavelengths (Figure 2A), and the differential desensitization of each produced by a high luminance yellow background [24,25]. Our ‘standard’ (named HFAm or modified Humphrey Field Analyzer) has been a commercially available automated perimeter using an add-on narrow band 440 nm interference filter, plus a bright yellow LED (light-emitting diode)-based background. The second instrument was an unmodified HFA (named HFAb) with blue on yellow (SWAP; short wavelength automated perimetry) settings used for glaucoma testing [22]. The third instrument was a MonCvONE (named MV for Metrovision). For this study, we configured the MV perimeter to use a narrowband filter identical to HFAm to allow the instrument agreement assessment between MV and our ‘standard’ HFAm. To be noted, the commercial versions of both the HFAb and MV perimeters use a wideband filter.

The background lights used in the three instruments have different spectral shapes but all were within the range of effective adaptation for the L/M-cone (and rod) mechanisms (Figure 2B). Across perimeters, the background effectiveness to adapt the L/M mechanism or the rods differed by less than 0.15 log units (l.u.) (photopic luminances of 123, 95, and 100 phot-cd.m^−2^; scotopic luminances of 40, 57, and 46 scot-cd.m^−2^ for HFAm, HFAb, and MV, respectively). All showed minimal overlap with the S-cone spectral sensitivity, preventing adaptation of this mechanism (with HFAm showing the least overlap because of the narrower spectrum of its LED background source).

Fitting the height of the S-cone and combined L/M-cone fundamentals to the low and high portion of data from the normal subjects (Figure 2C) resulted in a difference between the expected sensitivities of the two mechanisms at 440 nm of approximately 2 l.u. (green vertical line). The value of the difference, isolation, is consistent with previously reported estimates using a different method [24]. The 440 nm target on such backgrounds would be mediated by the S-cone mechanism in all cases in which its sensitivity reduction does not exceed 2 l.u. plus any L/M-cone sensitivity reduction. For example, if a patient shows 1 l.u. of L/M-cone sensitivity loss, the system can potentially be specific for S-cone measurements for S-cone losses up to 3 l.u. In practice, however, this range is reduced by the light intensity limitation of the target source that sets the instrument dynamic range.

On the high end of sensitivities (where the instrument uses dim light), the three instruments were found capable of measuring all sensitivities without evidence of a ceiling effect. *NR2E3*-ESCS patients can show higher than normal sensitivities, so in addition to data from the normal cohort we analyzed data from a cohort of *NR2E3*-ESCS patients (*n* = 37) [13]; we obtained the distributions of all observed sensitivity measurements. For both cohorts, the upper boundary for outlier detection by Tukey’s method (Q3 + 1.5 * interquartile range) [26] lies below the instrument upper limit range of 50 dB. This instrument limit therefore does not risk a ceiling artifact in either normal or *NR2E3*-ESCS patients, as values above this limit would be rarely expected to appear in practice.

The limiting factor for dynamic range is on the lower end of sensitivities, where bright light is required for the target. Wideband filters permit delivery of higher stimulation levels, extending the low-sensitivity dynamic range. Narrowband filters can provide better isolation but would require stronger light sources to equate the effective power content (Figure 2B, Stimuli). We evaluated the effect of filter choice as it impacts the dynamic range; the dynamic ranges in two instruments, the HFAm (narrowband filter) and the HFAb (wideband filter) were compared (Supplementary Figure S1A), and the HFAb did show a wider dynamic range allowing for the capture of lower-sensitivity measurements than the HFAm. We also studied the agreement between two different perimeters under comparable configurations for measurements of S-cone sensitivity (Supplementary Figure S1B). As the choice of target filter (narrowband or wideband) influences the range of sensitivity values, and this in turn has an effect on the pattern of light intensity presented by the threshold-finding algorithms and changes the floor effects limit, we decided to configure the two machines (HFAm and MV) with the same 440 nm narrowband filter for the agreement study. As the machines use different reference levels for their respective logarithmic scale (dB), there is a methodological bias added to the random fluctuation of the differences. We also looked at the variation of the bias with the level of the measurement and found it to be small (linear coefficient of 0.12 dB/dB, linear regression). We ignored this variation, as it would correspond to a departure no greater than 3.6 dB throughout the measurement range, and not considered clinically significant. The results indicate that the difference in measurements between the two machines (MV minus HFAm) is expected to be ±5.6 dB (95% confidence interval).

Our conclusion based on this instrumentation comparison is that the automated perimeters in their commercial setups (HFAb or MV) would be more advantageous and convenient to use in a clinical trial or natural history study of S-cone function in *NR2E3*-ESCS patients. In addition to a history of use in glaucoma testing, the wideband stimulus enables measuring less sensitive loci and this is important when assessing efficacy in regions of the field with diminished S-cone sensitivity.

### 2.2. Using S-cone Perimetry to Describe Disease Progression and Severity of NR2E3-ESCS Patients

**Figure 3:**
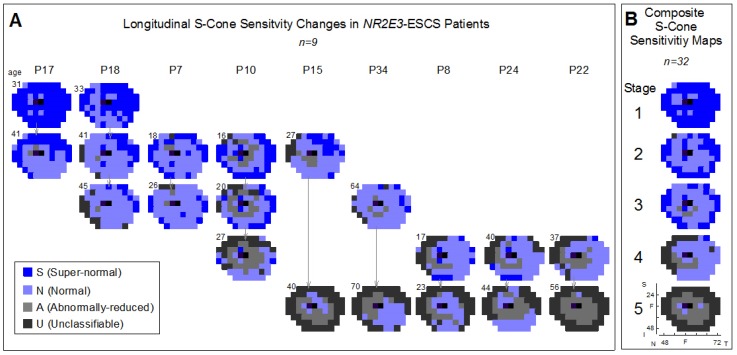
Progression of disease in *NR2E3*-ESCS. (**A**) S-cone sensitivity maps for patients who had multiple visits (*n* = 9) are arranged temporally (vertically; patient ages in upper left corner of map) for each patient and visually grouped (rows) based on patterns of S-cone function across the visual field (dark blue = super-normal sensitivity (S); light blue = normal sensitivity (N), light gray = abnormally-reduced sensitivity (A), dark gray = unclassifiable (U), cannot distinguish between A or N classification due to ‘floor effect’). Inspection of these longitudinal data leads to the proposal of 5 stages of disease progression in *NR2E3*-ESCS. (**B**) Composite S-cone sensitivity maps, generated from the aggregation of a large cohort of patient data (*n* = 32 patients), show the patterns and features of S-cone sensitivity that are characteristic of each of the 5 proposed disease stages. Horizontal and vertical axes in B, Stage 5 map, applies to all other maps in (**A**) and (**B**). N: nasal, T: temporal, I: inferior, S: superior, and F: fovea.

*NR2E3-*ESCS patients who were studied longitudinally have been previously reported [13]. We have added to this cohort and have now arranged the S-cone perimetry maps from the serial visits in columns to understand the progression of disease severity (Figure 3A). For example, P18 at age 33 years showed S-cone hypersensitivity over most of the temporal and superior visual field and normal S-cone function in remaining areas in the nasal and inferior fields. The amount of field occupied by hypersensitive S-cone function diminished by age 41 with only temporal superior islands remaining and a field that mainly was normal in S-cone sensitivity. At age 45, the field had both normal and subnormal S-cone function and a few superior-temporal loci of S-cone hypersensitivity. Other patients showed stages with less S-cone hypersensitivity progressing to S-cone ‘scotomas’ in the periphery. Using these serial data as guides, five disease stages were identified in a group of 32 patients with *NR2E3-*ESCS (Figure 3B). There were different numbers of patients in each stage: Stage 1, *n* = 4; Stage 2, *n* = 7; Stage 3, *n* = 10; Stage 4, *n* = 8; and Stage 5, *n* = 3. The average number of loci with S-cone hypersensitivity, normal sensitivity, reduced sensitivity and unclassifiable sensitivity were as follows: Stage 1—54/16/0/0; Stage 2—31/34/2/3; Stage 3—23/38/6/3; Stage 4—4/37/10/19; and Stage 5—0/3/26/41. Measurements affected by the floor effect were regarded as unclassifiable (whether or not they were normal or had abnormally-reduced sensitivities). 

The stages of the disease progression are to be interpreted as part of a continuum with variability in individual patients, yet modeling the generalized progression of the disease. In general, Stage 1 subjects have hypersensitive S-cone loci across the entire visual field, with some small areas of normal sensitivity. In Stage 2 of the disease, the number of normal S-cone loci increase relative to the hypersensitive loci, largely in the mid-periphery of the visual field; the hypersensitive S-cones are found further peripheral compared to what is observed in Stage 1. In Stages 2 and 3, a region of abnormally-reduced S-cones begins to form at a pericentral annulus extending from approximately 10−40° [13]. In Stage 3 of the disease, there are fewer hypersensitive loci than observed in Stage 2 and generally more normal and abnormally-reduced S-cones relative to hypersensitive loci. By Stage 4, hypersensitive loci are still present, but very scarce even in the far-periphery, and the field is dominated by loci having normal sensitivity, with the region of abnormality expanding further towards the periphery. Finally, in Stage 5, the entire field is mainly composed of abnormally-reduced S-cone sensitivities, with only a few normal regions remaining; hypersensitive loci are not observed and are instead replaced by regions of S-cone scotomas in the far periphery (Figure 3B).

### 2.3. Regional Variation of S-cone Function Across the Visual Field and Strategies for Monitoring Outcomes

**Figure 4:**
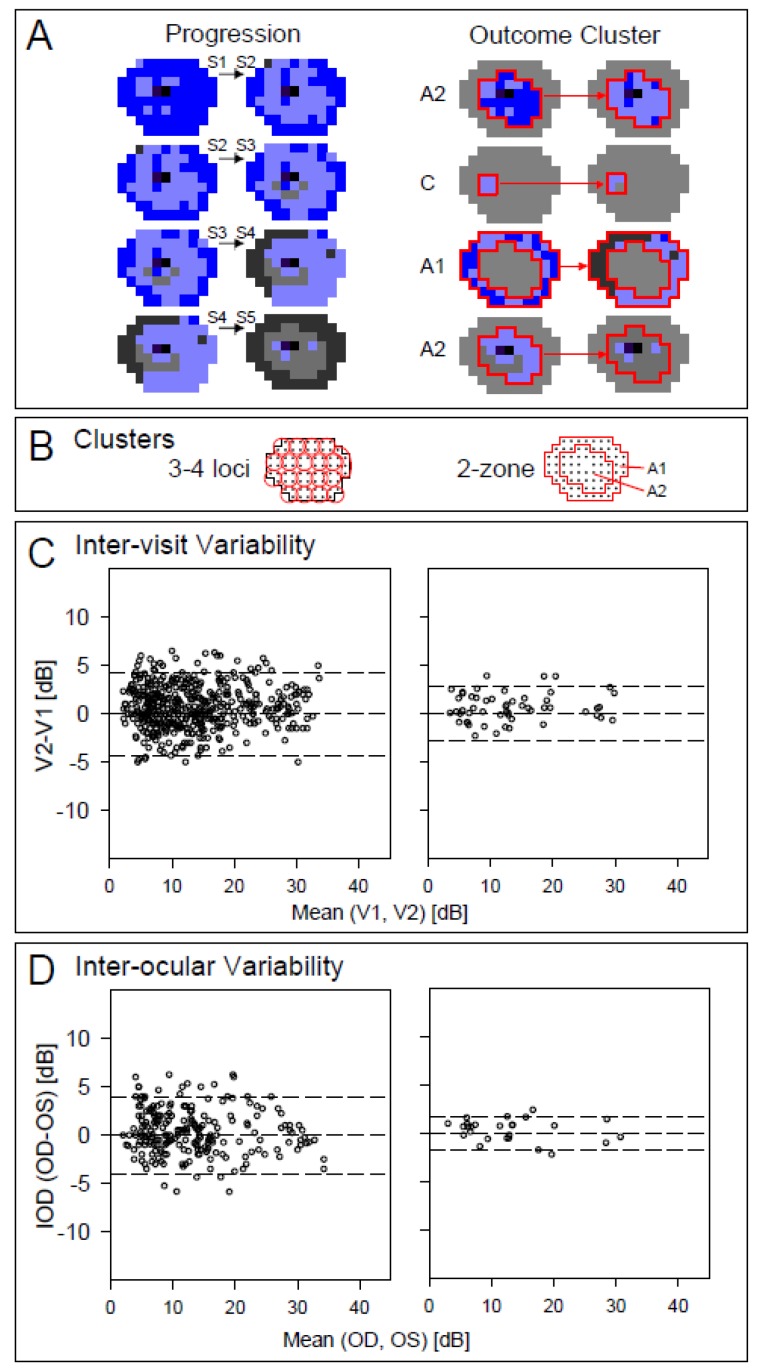
Analyzing change in S-cone perimetry as an outcome. (**A**) Left: maps illustrating progression from one stage of disease severity to the next in *NR2E3*-ESCS. Right: examples of strategies to measure efficacy outcomes for treatment at Stages 1–4 based on expected progression of disease: zones A1, A2, or one of the 3–4 loci clusters (C) were selected as they would likely predict the greatest changes at each stage. (**B**) Two aggregation (averaging) outcome strategies: by 3–4 loci clusters (*n* = 19 clusters, left) and by 2-zone (*n* = 2 rings, right). (**C**) Inter-visit variability (sensitivity at Visit2 minus Visit1) and (**D**) Inter-ocular (right eye minus left eye). Bias and variances do not appear correlated to the measurement level.

Unlike visual assays in other retinopathies, the unique disease mechanism in *NR2E3*-ESCS requires consideration of S-cone vision across the full visual field. Staging by S-cone perimetry at screening for a clinical trial would allow for decisions about inclusion and exclusion as well as how best to monitor for efficacy. Whether the outcome of a trial was expected to be visual improvement or slowing of disease progression, there would still be a need to understand which retinal regions could change in response to therapy. In the least complex of schemes, baseline (pre-treatment) could occur at stage *n* and the post-treatment trial outcome could be measured at stage *n* + 1. There are four possible stage pairs (Figure 4A, left) and for each pair we can determine which areas are expected to change most during the course of the trial. We present two clustering schemes for the data (Figure 4B): 3–4 loci clusters covering the entire visual field (resulting in a total of 19 clusters each incorporating 3–4 loci from the 12 ° grid test) and a 2-zone scheme (A1, far periphery and A2, pericentral).

Examples are shown of how the two clustering schemes could be used to distinguish differences in S-cone function at various stages (Figure 4A, right). For a trial starting at Stage 1, zone A2 (pericentral ring) would detect a change from a majority of hypersensitive S-cone loci to a majority with normal S-cone sensitivity. If a localized treatment (such as a subretinal injection) was delivered to the temporal retina (nasal field) in a patient at Stage 2, a small region (e.g., C) could be chosen for analysis while other regions not involved in the treatment could serve as controls. The third and fourth examples are for treatment starting at Stages 3 and 4, respectively. When starting in Stage 3, the A1 zone was chosen for treatment, and for Stage 4, zone A2 was chosen; maximum changes with progression would be expected in the peripheral (A1) zone for Stage 3 while in the pericentral zone (A2) for Stage 4.

The two clustering schemes for aggregating the individual location data were examined for inter-visit (Figure 4C) and inter-ocular (Figure 4D) variability in the normal subjects. For the 19-cluster set obtained by averaging 3–4 neighboring loci the 95% confidence interval for inter-visit differences was ±4.4 dB. The corresponding interval for the 2-zone set for assessment of the pericentral and peripheral was ±2.88 dB. Both strategies permit contrasts between treated and untreated areas for localized treatments such as sub-retinal injections, where the therapeutic agent reaches only a portion of the retina. The inter-ocular variability counterparts were ±4.1 and ±1.8 dB for 19 loci clusters and the 2-zone set, respectively.

### 2.4. Results Summary

The outcome measure for natural history studies and potential treatment trials of *NR2E3*-ESCS patients should involve monitoring of S-cone function. Chromatic adaptation permits S-cones to be isolated in the clinic through computerized static perimetry but the practical issues of availability and clinical feasibility of the measurement device must be considered. By comparing sensitivities on three automated static perimeters using spectral stimuli and chromatic backgrounds of varying intensity, we determined that S-cone function is indeed quantifiable in a cohort of normal subjects. The spectral sensitivity results matched previously published measurements for normal subjects [6]. Here we obtained estimates of the attainable isolation between the S- and L/M- cone mechanisms at testing conditions, and found it in agreement with other estimations of isolation obtained by a different methodology [24]. We verified that the outcome is S-cone specific throughout the attainable dynamic range. The dynamic ranges were also obtained and found to fit a relatively large *NR2E3*-ESCS patient population distribution without observing ceiling effects. Among the available instruments, an unmodified automated perimeter with a broad-band short-wavelength stimulus and bright yellow background would serve the purpose for a trial.

A staging of *NR2E3*-ESCS disease using S-cone perimetry was proposed and included five levels, which could be used to develop inclusion/exclusion criteria for clinical trials of this retinopathy. The staging would also be useful to define cohorts of patients in future trials. We studied the effect of clustering loci (using two methods) on both inter-visit and inter-ocular variability. The results of the current study provide useful steps toward clinical trials of treatment, whether focal or retina-wide, of patients with autosomal recessively inherited *NR2E3*-ESCS.

## 3. Materials and Methods

### 3.1. Subjects

Subjects with normal vision (*n* = 5; ages 22–34) were studied for the results in Section 2.1. For the retrospective data concerning the 37 patients with *NR2E3* mutations (ages 11–70), the normal subjects were age-related (*n* = 24; ages 19–56) [8]. The studies were in accordance with the tenets of the Declaration of Helsinki, and informed consents were obtained. All procedures were approved by the University of Pennsylvania institutional ethics review board; the ethical approval numbers of the studies are 183400 (26 July 1996 originally approved; 01 October 2018, renewed), 226100 (06 July 1995 originally approved; 15 May 2018 renewed) and 704353 (25 October 2001 originally approved; 11 October 2018 renewed).

### 3.2. Testing Procedures, Instrumentation, and Analysis

The measurement paradigm targets the short-wavelength cone (S-cone) mechanism by means of static perimetry [6,13,23,24]. In brief, the stimulus we used was 1.7° in diameter and 200 ms duration with spectral content centered at 440 nm on a yellow background with very low energy content below 530 nm. There were loci sampled on a 12° grid test as well as horizontal and vertical profiles at 2° intervals across the central 60° [23,27].

Acknowledging that all clinics do not have the same computerized perimeter, we studied three perimeters and compared their stimuli and backgrounds. The different spectral characteristics are shown (Figure 2). For the stimulus, the instruments use a filter to shape the spectrum of a white light source, with two types of filter (either narrowband or wideband). Our ‘standard’ (named for the present work as HFAm) has been used by us for almost 3 decades in research to characterize S-cone function across the visual field in normal subjects and retinal degeneration patients [6,8,23]. It is a commercially available automated perimeter (HFA-750i, Zeiss-Humphrey, Dublin, CA, USA) using an add-on narrowband 440 nm interference filter (10 nm full-width at half-maximum (FWHM), Ealing Electro-optics, Natick, MA. mod. 35-3345), plus a bright (123 cd/m^2^) yellow background from an external LED. We compared this research perimeter individually to two other designs available on the market: an unmodified HFA (named HFAb) with regular blue on yellow (SWAP; short-wavelength automated perimetry) settings used for glaucoma testing, and a MonCvONE (MetroVision, Perenchies, France) in a similar setup (named MV). The HFAb perimeter uses a wideband filter, and the MV perimeter was specially configured to use a narrowband filter identical to the research machine HFAm.

To verify the range of S-cone mediation of the stimulus before L/M intrusion occurs, we studied the relative spectral sensitivity characteristic of normal subjects under the testing conditions. We measured sensitivity at eight wavelengths over the range of 420–650 nm by inserting narrowband interference filters (7.0–11.5 nm FWHM) in the perimeter white beam. The spectral content of each beam was measured with a spectrometer to determine the energy content and make the spectral sensitivity measurements independent of the unequal intensities of the beams. The irradiance of each beam relative to the 500 nm beam was obtained by calculating the log of the fraction of the area under its spectral curve, relative to the area under the 500 nm-target curve. The relative irradiance (in l.u.) at each wavelength was algebraically added to the corresponding sensitivity (also in l.u.) to obtain the final subject spectral sensitivity values. A separate set of factors was used for each machine, as the light sources on the perimeters differ in spectral content. At least three measurements were obtained for each wavelength on the subjects’ preferred eye at locations 12°, 20°, and 36° in the inferior field. The variation of sensitivity versus wavelength was assessed as the wavelength-dependent component of a generalized additive model, controlling for location and perimeter, with subject as a random effect. We then manually adjusted the height of the S- and combined L/M-cone fundamentals [28] to the resulting wavelength-dependent model component (Figure 2C). The height adjustments were done to minimize differences over the 420–440 nm or 560–650 nm ranges for the S- and L/M-fundamentals, respectively. We obtained the amount of isolation for 440 nm targets by subtracting the ordinates of the two adjusted cone fundamentals evaluated at 440 nm (green vertical line).

To determine the dynamic range and variability of the S-cone perimetry outcome, sensitivities were measured at three sets of multiple loci: a full-field test with 76 loci on a 12° grid and vertical and horizontal profiles over the respective meridians, each with 30 loci separated by 2°. Each subject performed these three sets of tests on the three perimeters four times (both eyes tested on two different days) with the exception of one subject (the MV perimeter was unavailable). Measurements with zero values were excluded to control for floor effects. Agreement between HFAm and MV perimeter was assessed at the 95% level for subject, location and eye-specific differences between measurements in both machines (MV minus HFAm), using a mixed-effects model to allow for the correlation structure of the data. The model used Location as fixed effect and Subject and Eye as nested random effects [29]. We also included a linear term to test for proportional bias. The inter-visit variability was assessed using subject, location and eye-specific differences from two visits (Visit 2 minus Visit 1). Inter-ocular variability was likewise assessed using OD (right eye) minus OS (left eye) differences. The inter-visit and inter-ocular confidence interval assessments were obtained from mixed-effects models with Perimeter and Location as fixed effects, and either Subject and Eye or Subject and Visit as nested random effects, respectively. We used Tukey’s test (Q1 − 1.5 * IQR, Q3 + 1.5 * IQR) [26] to exclude possible outliers, separately for inter-visit and inter-ocular differences. Normality of residuals was inspected visually using quantile–quantile plots and found acceptable.

We obtained inter-visit and inter-ocular variability results for aggregated data from several neighboring loci as alternative measures to be considered in clinical trials. Two criteria for aggregation were used: first, we partitioned the 12° grid test in 19 approximately 24°-wide clusters homogeneously distributed throughout the field, each containing 3–4 original loci. Second, we defined two clusters partitioned by eccentricity to correspond to *NR2E3*-ESCS patterns [13]. These clusters contain 30 and 41 loci for pericentral and far peripheral field, respectively. For the aggregated analyses, data from loci within each cluster were averaged before proceeding with the variability assessments.

For all measurements, pupils of subjects were dilated and eyes adapted to the yellow background for five minutes before each test. Testing was performed under video monitoring of subjects’ eyes, with brief pauses to avoid fatigue. Absolute luminance measurements were performed with a research radiometer (IL1700, International Light, Peabody, MA, USA) with calibrated photopic and scotopic scales. Spectral irradiances were measured with a spectrometer (USB-2000, Ocean Optics, Largo, FL, USA) calibrated with LS-1 3100 K tungsten-halogen and HG-1 Hg–Ar sources (for relative irradiance and wavelength respectively).

### 3.3. Mapping Disease Severity of NR2E3-ESCS Patients Using S-cone Perimetry

We studied data from *NR2E3*-ESCS patients to understand disease progression. S-cone sensitivity maps were created to illustrate levels of function across the visual field for *NR2E3*-ESCS patients; for the patients, data were all collected with HFAm and the age range of patients led to the use of a normal data set ranging in age from 19–56 years [8]. Loci were classified according to levels of function as previously described [13]. Loci were either super-normal (S), abnormally-reduced (A), normal (N), or unclassifiable (U) in instances where there is a ‘floor effect’ whereby the lower limits of the instrument precludes the distinction between a normal and abnormally-reduced categorization. Super-normal and abnormally-reduced were defined as being greater than or less than the mean of normal ±2 SD at a given locus, respectively [13].

To determine how the disease progresses, we studied longitudinal S-cone sensitivity data. For patients with multiple visits (*n* = 9), S-cone sensitivity maps were arranged temporally and visually grouped based on patterns of S-cone function across the visual field; 5 general disease stages were identified. Composite maps were generated using a wider cohort of *NR2E3*-ESCS patients (*n* = 32 patients) to create generalized representations of the stages that show how patterns of S-cone function change across the visual field with time. Data collected during each patient’s first (or only) visit were used, so as to sample each patient once in generating the composites. Each individual map was visually categorized into one of the 5 disease stages. For each stage, a ‘mode’ rule was used to create the aggregated map: the S-cone classifier (S, A, N, or U) that appeared the most times at a given locus was assigned to that locus in the composite map. At loci where the mode could not be determined, the composite classifier was assigned based on the numerical average of the sensitivity measurements.

## Supplementary Materials

Supplementary materials can be found at https://www.mdpi.com/1422-0067/20/10/2497/s1.

## References

[1] Jacobson S.G., Cideciyan A.V., Sumaroka A., Roman A.J., Charng J., Lu M., Choi W., Sheplock R., Swider M., Kosyk M.S. (2017). Outcome measures for clinical trials of Leber congenital amaurosis caused by the intronic mutation in the CEP290 gene. Invest. Ophthalmol. Vis. Sci..

[2] Jacobson S.G., Cideciyan A.V., Sumaroka A., Roman A.J., Charng J., Lu M., Choudhury S., Schwartz S.B., Heon E., Fishman G.A. (2017). Defining outcomes for clinical trials of Leber congenital amaurosis caused by GUCY2D mutations. Am. J. Ophthalmol..

[3] Calzetti G., Levy R.A., Cideciyan A.V., Garafalo A.V., Roman A.J., Sumaroka A., Charng J., Heon E., Jacobson S.G. (2018). Efficacy outcome measures for clinical trials of USH2A caused by the common C.2299delG mutation. Am. J. Ophthalmol..

[4] Csaky K.G., Richman E.A., Ferris F.L. (2008). Report from the NEI/FDA Ophthalmic clinical trial design and endpoints symposium. Invest. Ophthalmol. Vis. Sci..

[5] Fishman G.A., Jampol L.M., Goldberg M.F. (1976). Diagnostic features of the Favre-Goldmann syndrome. Brit. J. Ophthalmol..

[6] Jacobson S.G., Marmor M.F., Kemp C.M., Knighton R.W. (1990). SWS (blue) cone hypersensitivity in a newly identified retinal degeneration. Invest. Ophthalmol. Vis. Sci..

[7] Marmor M.F., Jacobson S.G., Foerster M.H., Kellner U., Weleber R.G. (1990). Diagnostic clinical findings of a new syndrome with night blindness, maculopathy, and enhanced S cone sensitivity. Am. J. Ophthalmol..

[8] Jacobson S.G., Roman A.J., Roman M.I., Gass J.D., Parker J.A. (1991). Relatively enhanced S cone function in the Goldmann-Favre syndrome. Am. J. Ophthalmol..

[9] Hood D.C., Cideciyan A.V., Roman A.J., Jacobson S.G. (1995). Enhanced S cone syndrome: Evidence for an abnormally large number of S cones. Vision. Res..

[10] Milam A.H., Rose L., Cideciyan A.V., Barakat M.R., Tang W.X., Gupta N., Aleman T.S., Wright A.F., Stone E.M., Sheffield V.C. (2002). The nuclear receptor NR2E3 plays a role in human retinal photoreceptor differentiation and degeneration. Proc. Natl. Acad. Sci. USA.

[11] Haider N.B., Mollema N., Gaule M., Yang Y., Sachs A.J., Nuysten A.M., Naggert J.K., Nishina P.M. (2009). Nr2e3-directed transcriptional regulation of genes involved in photoreceptor development and cell-type specific phototransduction. Exp. Eye Res..

[12] Wright A.F., Reddick A.C., Schwartz S.B., Ferguson J.S., Aleman T.S., Kellner U., Jurklies B., Schuster A., Zrenner E., Wissinger B. (2004). Mutation analysis of NR2E3 and NRL genes in enhanced S Cone syndrome. Hum. Mutat..

[13] Garafalo A.V., Calzetti G., Cideciyan A.V., Roman A.J., Saxena S., Sumaroka A., Choi W., Wright A.F., Jacobson S.G. (2018). Cone vision changes in the enhanced S-cone syndrome caused by NR2E3 gene mutations. Invest. Ophthalmol. Vis. Sci..

[14] Mollema N.J., Yuan Y., Jelcick A.S., Sachs A.J., von Alpen D., Schorderet D., Escher P., Haider N.B. (2011). Nuclear receptor Rev-erb Alpha (Nr1d1) functions in concert with Nr2e3 to regulate transcriptional networks in the retina. PLoS ONE.

[15] Swaroop A., Kim D., Forrest D. (2010). Transcriptional regulation of photoreceptor development and homeostasis in the mammalian retina. Nat. Rev. Neurosci..

[16] Kim J.W., Yang H.J., Brooks M.J., Zelinger L., Karakülah G., Gotoh N., Boleda A., Gieser L., Giuste F., Whitaker D.T. (2016). NRL-regulated transcriptome dynamics of developing rod photoreceptors. Cell. Rep..

[17] Cruz N.M., Yuan Y., Leehy B.D., Baid R., Kompella U., DeAngelis M.M., Escher P., Haider N.B. (2014). Modifier genes as therapeutics: The nuclear hormone receptor Rev Erb alpha (Nr1d1) rescues Nr2e3 associated retinal disease. PLoS ONE.

[18] Rabin J.C., Kryder A.C., Lam D. (2016). Diagnosis of normal and abnormal color vision with cone-specific VEPs. Transl. Vis. Sci. Technol..

[19] Huchzermeyer C., Kremers J. (2016). Perifoveal L- and M-cone-driven temporal contrast sensitivities at different retinal illuminances. J. Opt. Soc. Am. A Opt. Image Sci. Vis..

[20] Stiles W.S. (1939). The directional sensitivity of the retina and the spectral sensitivities of the rods and cones. Proc. Royal Soc. Lond.

[21] Sample P.A., Weinreb R.N. (1990). Color perimetry for assessment of primary open-angle glaucoma. Invest. Ophthalmol. Vis. Sci..

[22] Johnson C.A. (1996). Diagnostic value of short-wavelength automated perimetry. Curr. Opin. Ophthalmol..

[23] Jacobson S.G., Voigt W.J., Parel J.M., Apathy P.P., Nghiem-Phu L., Myers S.W., Patella V.M. (1986). Automated light- and dark-adapted perimetry for evaluating retinitis pigmentosa. Ophthalmology..

[24] Sample P.A., Johnson C.A., Haegerstrom-Portnoy G., Adams A.J. (1996). Optimum parameters for short wavelength automated perimetry. J. Glaucoma..

[25] Demirel S., Johnson C.A. (2000). Isolation of short-wavelength sensitive mechanisms in normal and glaucomatous visual field regions. J. Glaucoma..

[26] Tukey J.W. (1977). Exploratory Data Analysis.

[27] McGuigan D.B., Heon E., Cideciyan A.V., Ratnapriya R., Lu M., Sumaroka A., Roman A.J., Batmanabane V., Garafalo A.V., Stone E.M. (2017). EYS mutations causing autosomal recessive retinitis pigmentosa: Changes of retinal structure and function with disease progression. Genes.

[28] Wyzecki G., Stiles W.S. (1982). Concepts and methods, quantitative data and formulae. Color Science.

[29] Parker R.A., Weir C.J., Rubio N., Rabinovich R., Pinnock H., Hanley J., McCloughan L., Drost E.M., Mantoani L.C., MacNee W. (2016). Application of mixed effects limits of agreement in the presence of multiple sources of variability: Exemplar from the comparison of several devices to measure respiratory rate in COPD patients. PLoS ONE.

