# Supplementary material for: Short-Wavelength Sensitive Cone (S-cone) Testing as an Outcome Measure for NR2E3 Clinical Treatment Trials
Source: Int J Mol Sci. 2019 May 21;20(10):2497. doi: 10.3390/ijms20102497 (PMC6566804; doi:10.3390/ijms20102497)
Supplement: Supplementary file 1 [file ijms-20-02497-s001.pdf]

SUPPLEMENTARY MATERIAL

Short-wavelength Sensitive Cone (S-cone) Testing as an Outcome Measure for NR2E3 Clnical Treatment Trials

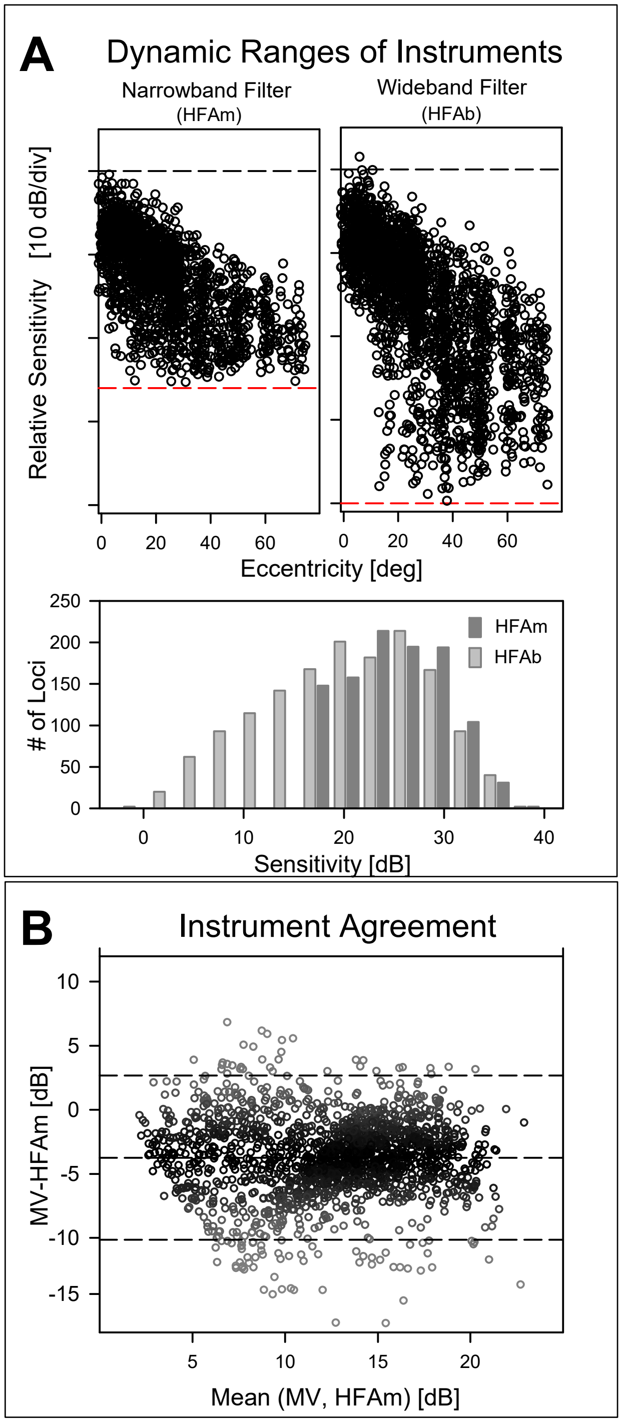

Supplementary Figure S1. Instrumentation dynamic ranges and agreement. (**a**) Sensitivity values obtained from the normal subjects on Machine HFAm and HFAb. The brighter stimuli in HFAb, due to the use of a wideband filter, permits measurement over an extended range, about 15 dB wider than that on machine HFAm (upper graphs). This is also evident in the absence of bars on the HFAm histogram over that range (lower). (**b**) Agreement between HFAm and MV instruments, both using a narrowband filter. There is a systematic bias of 2 dB due to the different light source intensities between instruments. Both bias and variance are not strongly correlated to measurement level.
